# Impact of Concurrent Use of N95 and Surgical Masks on Peripheral Oxygen Saturation and Heart Rate Frequency—A Prospective Study during the COVID-19 Outbreak

**DOI:** 10.3390/medicina60020276

**Published:** 2024-02-05

**Authors:** Anna Alessandri-Bonetti, Linda Sangalli, Patrizia Gallenzi

**Affiliations:** 1 Department of Dental Clinic and Maxillofacial Surgery, A. Gemelli University Policlinic IRCCS, Catholic University of Sacred Heart, Largo Francesco Vito 1, 00168 Rome, Italy; anna.alessandribonetti01@icatt.it (A.A.-B.); patrizia.gallenzi@unicatt.it (P.G.); 2 College of Dental Medicine—Illinois, Midwestern University, 555 31st Street, Downers Grove, IL 60515, USA

**Keywords:** N95, COVID-19 pandemic, oxygen saturation, heart rate, surgical facemask, dental providers

## Abstract

*Background and Objectives*: The COVID-19 outbreak has necessitated the prolonged use of N95 facemasks in addition to traditional surgical facemasks by healthcare workers. The aim of this study was to investigate the effect of wearing N95 facemasks in addition to surgical facemasks on peripheral oxygen saturation (SpO_2_) and heart rate (HR) among dental professionals during routine care. *Materials and Methods*: This prospective study compared SpO_2_ and HR between dental providers wearing N95 + surgical facemasks vs. wearing a surgical facemask only. SpO_2_ and HR were recorded using a portable pulse oximeter before wearing the facemask (T0); at 30 min (T1); at 1 h (T2); and at the end of clinical activity (T3). Inter-group and intra-group differences were assessed with independent *t* tests and repeated measures ANOVA, respectively. *Results*: A total of 88 participants (57 wearing N95 + surgical facemasks, and 31 wearing a surgical facemask only) completed the study. The two groups did not statistically differ in SpO_2_ at different timepoints nor showed any intra-group differences. The participants wearing N95 + surgical facemasks exhibited a statistically higher HR at T0 (*p* = 0.007), T2 (*p* = 0.010), and T3 (*p* = 0.014) compared to those wearing a surgical facemask only. A statistically significant decrease was observed in HR between T0 and T3 in those wearing N95 + surgical facemasks (*p* = 0.012). No intra-group differences were seen in HR over time in those wearing a surgical facemask only. *Conclusions:* The continuous use of an N95 in addition to a surgical facemask did not show any significant effects in SpO_2_ during routine care; however, the concurrent use of an N95 and a surgical facemask seemed to be accompanied by a decrease in HR, although the values remained within the normal range.

## 1. Introduction

The novel coronavirus, known as SARS-CoV-2, is the cause of the COVID-19 disease [1,2]. Since COVID-19 primarily spreads through respiratory droplets [3], the use of Personal Protective Equipment (PPE) such as facemasks with varying levels of filtration efficiency have been recommended worldwide for the general public [4,5] and especially for healthcare professionals. Among healthcare workers, dental providers have been appointed from the beginning of the pandemic as being at particularly high risk, due to the use of drills or ultrasonic units characterizing Aerosol-Generating Procedures (AGPs). As a result, healthcare workers including dental practitioners have been advised to incorporate an N95 alongside or in replacement of traditional surgical facemasks to enhance their protection against viral transmission [6,7]. The World Health Organization (WHO) suggests utilizing particulate respirators with a minimum equivalence to an N95 when conducting AGPs. However, for regular care involving infection prevention, the WHO deems type IIR fluid-resistant surgical masks to be adequate and sufficient [8]. In addition, dental professionals have been advised to utilize additional barrier-protection equipment such as protective eyewear, masks, gloves, caps, face shields, and protective gowns, due to the direct exposure to patients’ saliva [9,10]. In recent times, alongside the ongoing COVID-19 outbreak, a new outbreak of Monkeypox, a viral infection, has been identified. Also in this circumstance, new findings have highlighted the potential transmission of viruses through respiratory droplets [11], with evidence showing that the craniofacial region is often affected [12,13]. These observations reinforce the critical need for healthcare personnel to consistently utilize PPE.

The suggestion, and at times the requirement, to use concurrent PPE has been recognized as burdensome and occasionally associated with adverse effects [14]. Despite recommendations, many dental providers have opted for the use of surgical masks alone while performing dental procedures. This scenario has occurred in many dental healthcare settings, including our hospital. For example, despite the strong recommendation to wear an N95 in addition to surgical masks during all clinical activities regardless of AGP or not, many dental providers in our hospital consistently wore type IIR fluid-resistant surgical facemasks for those procedures that do not generate aerosols. Beside the different levels of protection from viral transmission and the subjective experience of fatigue, there is a gap in understanding the objective physiological effects of using concurrent PPE. As such, in this regard, earlier reports suggested that the use of concomitant PPE or N95 respirators might reduce peripheral oxygen availability and impede carbon dioxide exchange, thus potentially aggravating certain conditions [15,16]. Specifically, recent investigations examining the impact of facemasks on dental healthcare providers have shown a decrease in oxygen saturation (SpO_2_) levels while using an N95 [17,18]. However, these studies are limited by the lack of a control group and various methodological constraints. Conversely, another study investigating the impact of facemasks revealed an alteration in heart rate (HR) but failed to demonstrate any effect on oxygen saturation [19]. These contradictory findings highlight the importance of conducting well-designed studies to shed light on this subject.

Given the current gap in well-designed clinical studies evaluating the concurrent use of facemasks on physiological measures, the aim of the present study was to examine the effect of an N95 in addition to surgical facemasks on both SpO_2_ and HR in a population of dental professionals during routine care.

The hypothesis was that the participants wearing an N95 in addition to surgical facemasks will not experience a reduction in SpO_2_ or a change in HR. The findings of this study are expected to contribute valuable insights to the ongoing debate on the physiological impact of PPT use in healthcare settings.

## 2. Materials and Methods

### 2.1. Study Design

This unblinded, prospective cohort study was conducted at a large hospital setting (A. Gemelli Hospital, Rome, Italy) and recruited participants over a seven-month period (from January 2022 to July 2022). The study was approved by the Ethics Committee of the university where it was conducted and was in accordance with the STROBE guidelines. The protocol was registered on clinicaltrials.gov (NCT05188651). 

### 2.2. Sample Size Calculation

A sample size calculation was performed using a decrease in SpO_2_ by 3% or more as the clinically significant cutoff [20]. Based on a change of SpO_2_ of 3% and a standard deviation (SD) = 3 [21] to detect a statistically significant difference for *α =* 0.05 and power at 80%, the minimum sample size was calculated to be N = 31 participants in total.

### 2.3. Participants

Participants were recruited among all the dental professionals actively practicing at the A. Gemelli Hospital where the study was conducted, regardless of their appointment or status (dental students, residents, faculty members, attendings, dental assistants, and/or staff).

To be eligible, participants had to (1) be a dental provider currently practicing at the A. Gemelli Hospital, (2) be ≥18 years old, and (3) consent to partake in the study. Exclusion criteria were those subjects unable to wear a facemask due to an underlying condition (e.g., individuals with chronic respiratory problems for whom wearing a mask could impede breathing, disability, facial trauma, or any recent facial surgery).

### 2.4. Procedures

All participants signed a written informed consent and agreed to participate in the study. As explained above, some dental professionals decided to wear type IIR fluid-resistant surgical facemasks during dental procedures not generating aerosols. As a result, two groups were created. The first group included participants wearing N95 respirators in addition to surgical facemasks; the second group included those wearing only type IIR fluid-resistant surgical facemasks. All participants belonging to the first group were instructed to first wear an N95 and then a surgical facemask over it. All participants were instructed by the study personnel on how to correctly wear the facemask (i.e., ensuring complete coverage of the nose and mouth) before the beginning of the study and were provided with opportunities to ask for clarification [21].

### 2.5. Outcome Measures

The following physiological measurements were recorded using a portable pulse oximeter, clipped onto the right index finger:

- oxygen saturation (SpO_2_): peripheral capillary oxygen saturation is a measure of the percentage of oxygen-saturated hemoglobin in the bloodstream. It is used to assess respiratory function and oxygenation status. Normal values range between 95 and 100% [22].
- heart rate (HR): this refers to the number of times the heart beats per minute (beats per minute, bpm). It provides information on cardiac function and the overall cardiovascular health. Normal values range between 60 and 100 bpm [23].

### 2.6. Collection Timepoint

SpO_2_ and HR were recorded at four timepoints during a regular day of clinical activity. All measurements were taken on the same day and participants were recruited only once during the study period.

Participants wearing both N95 and surgical facemasks had their SpO_2_ and HR recorded as follows: while wearing a surgical facemask only; before wearing the N95 (T0); after 30 min using an N95 in addition to a surgical facemask (T1); after one hour wearing both facemasks (T2); and at the end of the clinical activity, after at least 4 h wearing both facemasks (T3).

**Figure 1:**
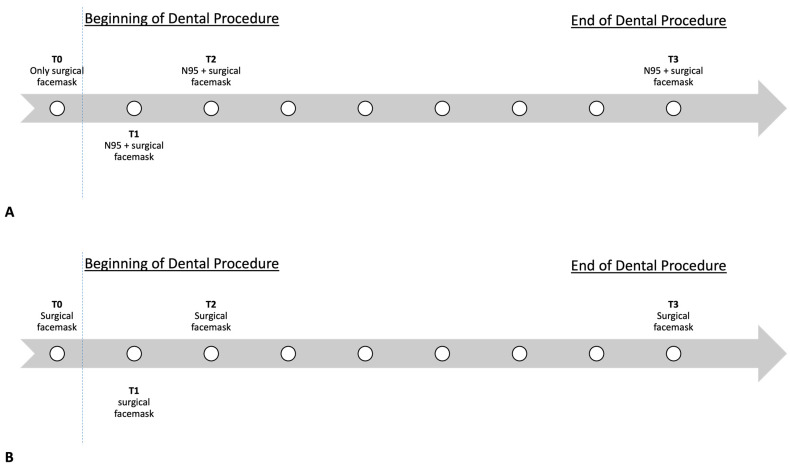
Details of the study procedure for participants wearing an N95 in addition to a surgical facemask (**A**) and participants wearing surgical facemask only (**B**). HR: heart rate; SpO_2_: oxygen saturation; T0: baseline, before starting the dental procedure; T1: at 30 min; T2: at 1 h; T3: at the end of the clinical activity (≥4 h). The dotted line represents the beginning of the dental procedure.

Participants wearing only surgical facemasks had their SpO_2_ and HR recorded at the same timepoints, with T0 being the measurement taken before starting any dental procedures while wearing the surgical facemask only. At each timepoint, two measurements were performed within 5 min and the mean value of the two was recorded. Measurement of SpO_2_ was performed over 15 s each time. Figure 1 displays the timeline of the study procedure.

### 2.7. Statistical Analysis

The normality of the data distribution was verified with the Shapiro–Wilk test, and the assumption of sphericity was assessed with Mauchly’s test of sphericity. For descriptive purposes, means, standard deviations, median, and the observed range were computed, as appropriate. Intra-group differences in SpO_2_ and HR were assessed at different timepoints using a repeated measures, mixed-model ANOVA. Significant *p* values were investigated through pairwise comparisons with Bonferroni adjustment as post hoc analysis. Next, differences between the two groups (N95 + surgical mask vs. surgical mask only) in SpO_2_ and HR were assessed using a Student’s *t* test at each timepoint.

For all analyses, the *p*-value was set at <0.05. Data were analyzed with statistical software SPSS (IBM SPSS Statistics for Macintosh, Version 27.0000, IBM Corp., Armonk, NY, USA) and graphical representations were generated with SAS studio (SAS Software, Copyright © 2023, SAS Institute Inc., Cary, NC, USA).

## 3. Results

A total of 117 people working at the dental clinic were eligible; however, only 88 participants (75.21%) were invited to participate in the study. This decision was due to differences in the schedule that would not allow for all measurements to be recorded (i.e., a schedule duration of less than 4 h). All invited participants accepted the invitation and none of them withdrew from the study. The final sample included 57 dental students, 17 dental residents, 10 dental assistants, 2 attendings, 2 faculty members, and 0 staff members. A total of 57 participants (mean age 28.9 ± 10.1, 50.9% males, 29.8% smokers) used an N95 in addition to a surgical facemask for all procedures, while 31 participants (mean age 30.9 ± 9.5, 32.3% males, 25.8% smokers). Four participants in the N95 group (7.0%) and four in the surgical mask group (12.9%) had at least one medical condition (controlled type 2 diabetes).

### 3.1. Intra-Group Differences in SpO_2_

Table 1 shows the pooled mean SpO_2_ in the two groups at different timepoints.

Among those wearing N95 + surgical facemasks, no participants’ SpO_2_ level dropped below 95%. A repeated measures ANOVA conducted among the surgical facemask group revealed that the mean SpO_2_ did not statistically significantly differ between any timepoints (F(3, 90) = 0.500, *p* = 0.68).

Among those wearing the surgical facemask only, no participants’ SpO_2_ level dropped below 96%. A repeated measures ANOVA conducted among the N95 + surgical facemask group revealed that the mean SpO_2_ did not statistically significantly differ between any timepoints (F(3, 168) = 2.404, *p* = 0.069).

### 3.2. Intra-Group Differences in HR

Table 2 shows the pooled mean HR in the two groups at different timepoints. A repeated measures ANOVA among those participants wearing an N95 + surgical mask revealed that the mean HR statistically significantly differed between time points (F(3, 168) = 3.774, *p* = 0.012). Post hoc analysis with Bonferroni adjustment revealed that the mean HR statistically significantly decreased from T0 to T3 (4.175 (95% CI 0.645, 7.706), *p* = 0.012), but not at any other timepoints.

**Figure 2:**
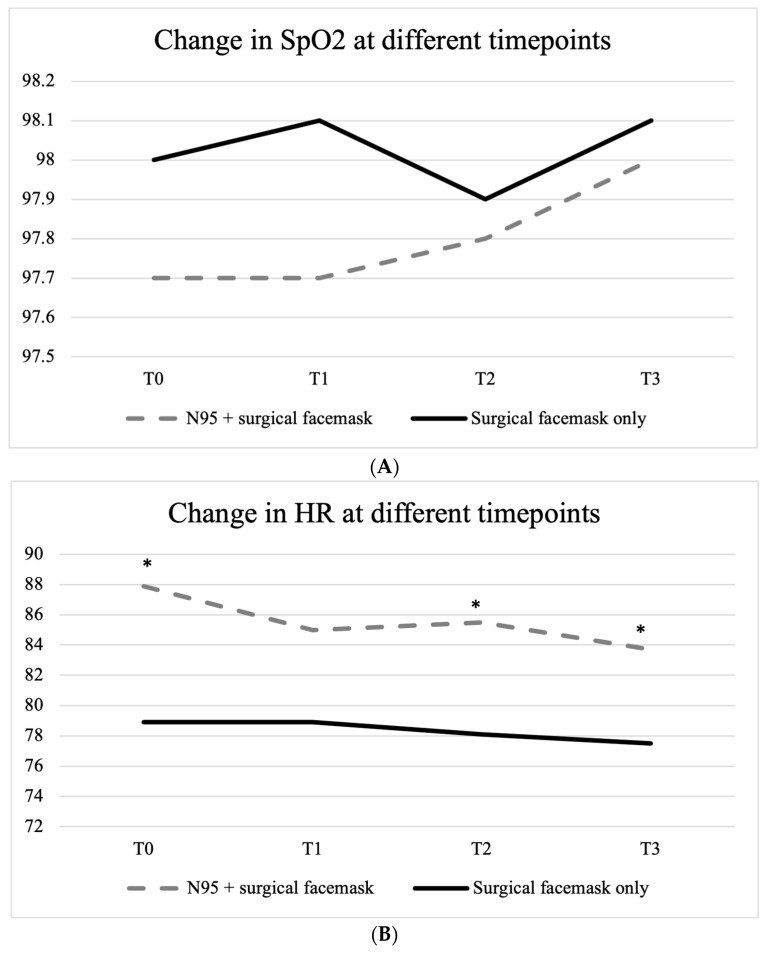
Change in SpO_2_ (**A**) and HR (**B**) at different timepoints. * denotes a statistically significant difference.

A repeated measures ANOVA among the participants wearing only a surgical facemask revealed that the mean HR did not statistically significantly differ between timepoints (F(3, 90) = 0.443, *p* = 0.72, Figure 2).

### 3.3. Inter-Group Differences in SpO_2_

**Figure 3:**
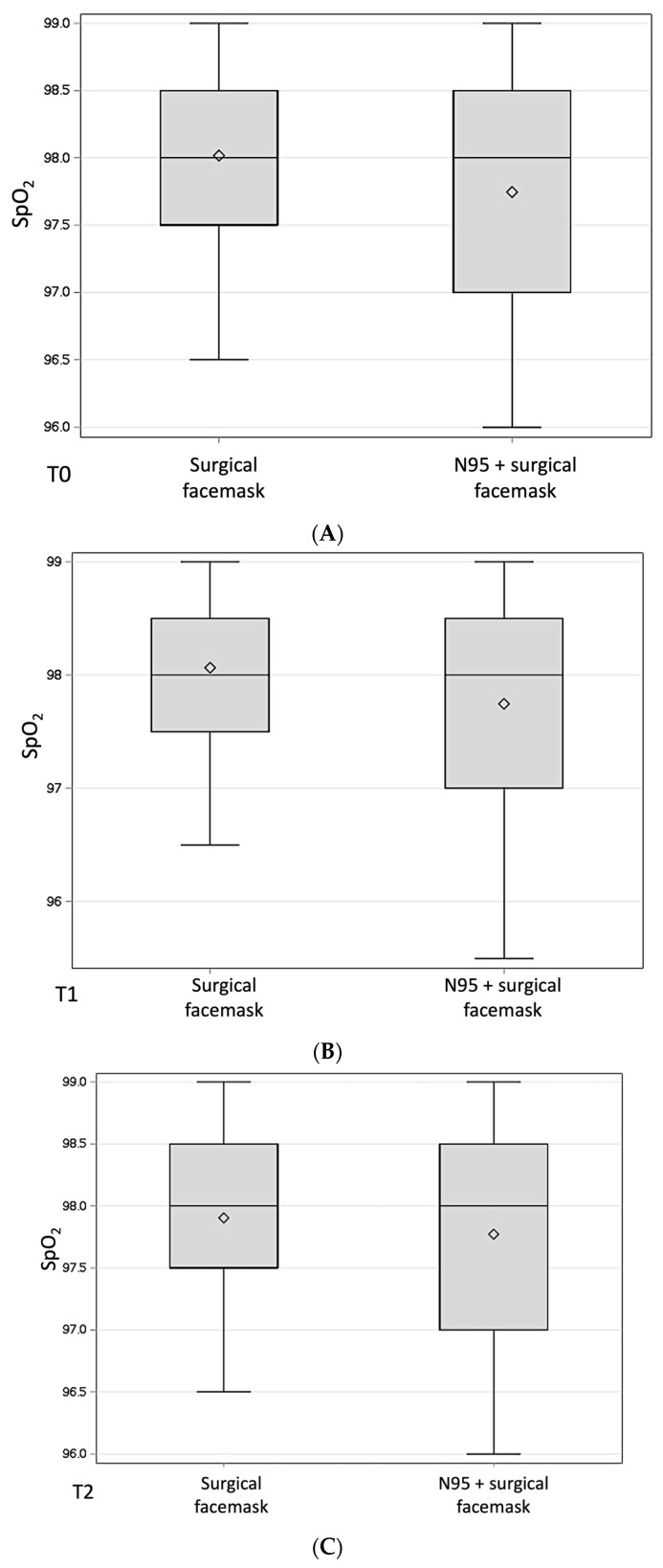

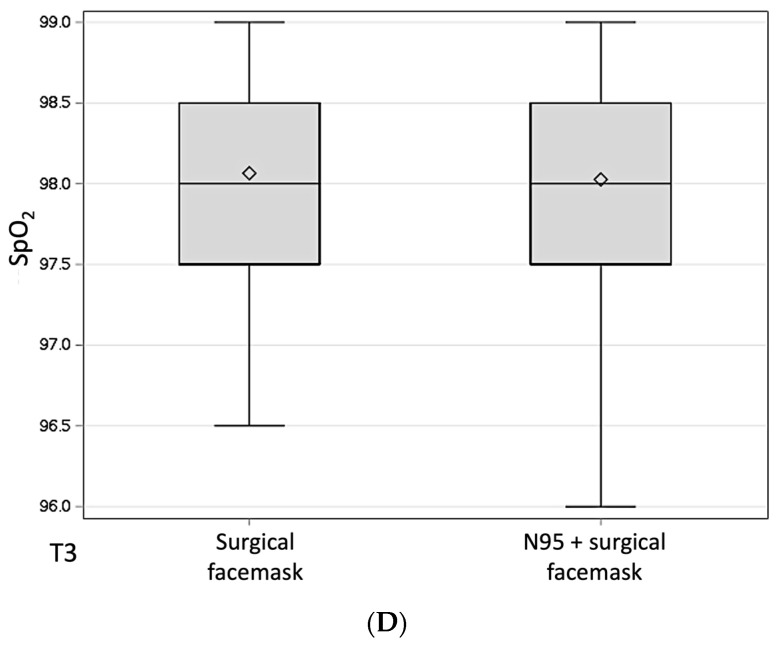
Boxplots displaying the difference in SpO_2_ (%) between participants wearing an N95 in addition to a surgical facemask and those wearing only a surgical facemask at T0 (**A**), T1 (**B**), T2 (**C**), and T3 (**D**). SpO_2_: oxygen saturation; T0: baseline, before starting the dental procedure; T1: at 30 min; T2: at 1 h; T3: at the end of the clinical activity (≥4 h).

No statistically significant difference was found at each timepoint in SpO_2_ between those wearing an N95 + surgical facemask and those only wearing a surgical facemask (Figure 3).

### 3.4. Inter-Group Differences in HR

**Figure 4:**
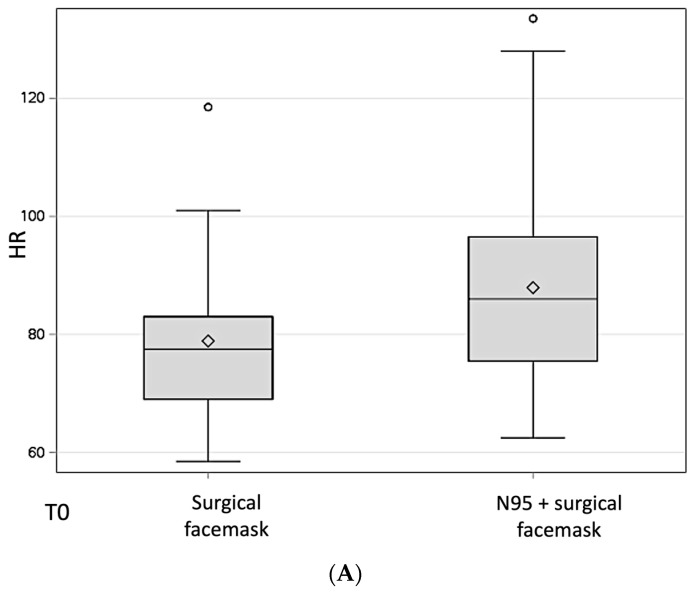

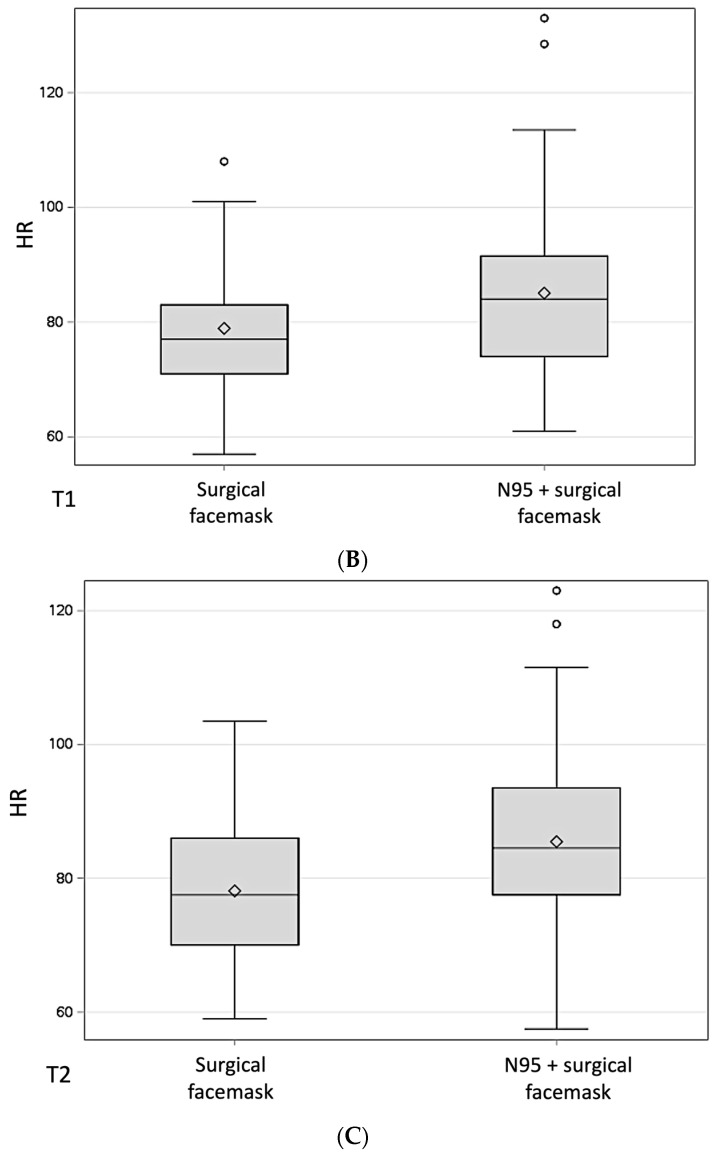

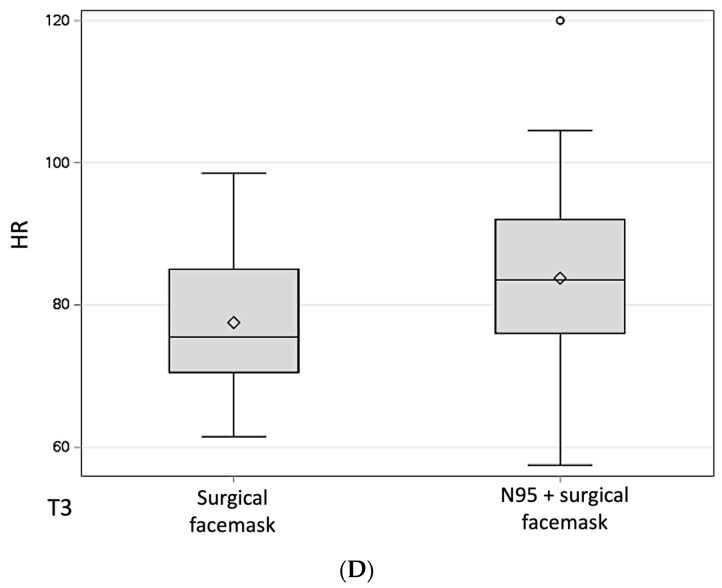
Boxplots displaying the difference in HR between participants wearing an N95 in addition to a surgical facemask and those wearing only a surgical facemask at T0 (**A**), T1 (**B**), T2 (**C**) and T3 (**D**). HR: heart rate; T0: baseline, before starting the dental procedure; T1: at 30 min; T2: at 1 h; T3: at the end of the clinical activity (≥4 h).

There was a statistically significant difference in HR between those wearing an N95 + surgical facemask and those only wearing a surgical facemask (Figure 2). Specifically, those wearing an N95 + surgical facemask had a significantly higher HR at T0 (87.9 ± 15.1 vs. 78.9 ± 13.6, *t* = 2.769, *p* = 0.007), at T2 (85.5 ± 13.7 vs. 78.1 ± 10.2, *t* = 2.618, *p* = 0.010), and at T3 (83.7 ± 11.9 vs. 77.5 ± 9.5, *t* = 2.504, *p* = 0.014), with all the 95% CIs of the mean differences not containing 0 (Table 2, Figure 4).

## 4. Discussion

Since the beginning of the COVID-19 outbreak, facemask use has become mandatory in many clinical settings worldwide, prompting investigations into their impact on healthcare workers. The aim of the present study was to observe the effect of wearing an N95 in addition to a surgical facemask, in comparison to wearing a surgical facemask only on SpO_2_ and HR in dental professionals during routine care. The hypothesis, therefore, was that no reduction in SpO_2_ and no variation in HR would be observed in dental workers concomitantly wearing an N95 in addition to a surgical facemask during routine care at different time points when compared to participants wearing a surgical facemask only.

As concerns SpO_2_, a lack of statistically significant difference was observed in physiological measurements taken among dental professionals, regardless of the facemask used and the time interval examined. Despite the SpO_2_ level fluctuating over time in both groups, the change at different timepoints ranged within 1% in both groups, thus was not considered statistically or clinically significant. These findings are concordant with those by Nwosu et al., which have shown no significant differences in the mean SpO_2_ in other healthcare workers irrespective of the type of facemask worn (an N95, surgical mask, or cloth mask) when comparing before and after surgical sessions [24]. Other authors have reported a reduction in SpO_2_ in healthcare workers wearing different types of facemasks [17,25]. Another study was conducted on 20 dental providers and revealed a reduction in SpO_2,_ comprising between 3.5% and 6.5%, at the end of the dental procedure compared to the initial measurements [17]. However, the length of the dental procedures varied significantly, lasting anywhere from 20 min to 240 min (~4 h). Moreover, only 4 participants were tested after 240 min, leading to an increased measurement variability and a smaller sample size. A second study by Beder et al. was carried out on 53 surgeons and revealed an SpO_2_ decrease after the first hour, especially in those participants older than 35 [25]. As the population of our study was, on average, younger (28.9 ± 10.1) than the one of Beder et al., different results cannot be excluded when compared to an older cohort of participants. Moreover, the lack of consistency between the results in the literature could be attributed to several factors, such as variations in the workload associated with surgical procedures [25], limited sample size, or different timeframe examined. Lack of consistency may also be related to an intra-individual physiological variability, or the reliability of the device being used. The pulse oximeter has an accuracy in detecting SpO_2_ of ±2% [26], thus a variation of this magnitude should not be considered clinically relevant, despite being potentially statistically significant. To the best of our knowledge, the present study is the first to examine SpO_2_ on the same participants at different timepoints and to compare data between participants wearing both N95 and surgical facemasks and those wearing surgical masks only. The findings of this study, which demonstrate no change in SpO_2_, offer evidence to support that protective facemasks do not cause respiratory impairment. These findings not only add scientific validity to anecdotal observations derived from clinical practice, but also underscore the reassurance that facemasks do not compromise oxygen levels. Additionally, the impact of surgical masks on neuro-electrical brain activity has been investigated in the general population, showing that facemasks do not influence this aspect [27].

As concerns HR, our data indicate mean values to be around 81.95 bpm. Despite the fluctuation observed at different time points, mean values remained within the range of the average healthy resting HRs of 60–100 bpm, as defined by the American Heart Association [23]. Interestingly, the measurement taken at T0 in the two groups already showed a statistically significant difference in that the participants wearing an N95 + surgical mask had a significantly higher HR (87.9 ± 15.1) compared to those wearing only a surgical facemask (78.9 ± 13.6). However, T0 coincided with the baseline measurement, i.e., with the participants wearing only a surgical mask with no additional N95 before starting any dental procedures. This means that the difference could not be adduced to the use of an N95 mask. To explain this finding, it is possible that the participants could experience an anticipatory stress response, related to the upcoming dental procedure or other situational factors. Stress is indeed known to elicit physiological responses, including an increase in HR. Accordingly, the participants may have experienced heightened anxiety or stress levels, leading to an elevated HR at the baseline measurement. However, since no data were collected on the types of procedure that the participants would perform, and considering that no psychological evaluation was performed, this remains a speculation and caution should be taken when drawing definite conclusions regarding the specific cause of the initial HR difference. Future research incorporating psychological assessments could help elucidate the underlying factors contributing to this observation. Another interesting finding was the significant reduction in HR (by 4 bpm) that the group wearing an N95 + surgical facemask experienced over time, although the value at T3 was still significantly higher compared to those wearing a surgical facemask only. This reduction, although statistically significant, might be of limited clinical significance since the values remained within the normal range. Contradictory results derived from the literature. While some studies in the literature have shown no difference in HR in healthcare workers after a 1 h treadmill walking session while wearing an N95 [28], others reported an increase in HR at the end of the surgical procedures [25]. Given the variability observed in the existing literature, further well-designed studies are needed to validate and elucidate the factors influencing HR fluctuations during mask use in healthcare professionals.

### Limitations

Some limitations should be acknowledged. The first shortcoming is the lack of randomization of the participants. As such, those who self-selected to wear an N95 in addition to a surgical mask may differ from those who decided to wear a surgical mask alone. Moreover, due to a lack of randomization, the value of HR at baseline was significantly different between the two groups. For ethical reasons, it was not possible to randomize the participants and future studies are needed to confirm the present findings. Similarly, another significant limitation is the lack of a control group not wearing masks, a constraint imposed by the need for homogeneity in the types of dental interventions performed by the participants. Another shortcoming is the relatively young age of the participants that limits the generalizability of the findings, given that elderly professionals may experience a more significant fluctuation in SpO_2_ [23]. Nevertheless, none of the studies conducted in an elderly population have so far observed any significant fluctuation in SpO_2_ [21]. An additional limitation arises from the different sealing capabilities of surgical facemasks and type IIR fluid-resistant surgical facemasks, potentially impacting the accuracy of SpO_2_ readings. This aspect highlights a practical consideration that warrants attention in future research, where the choice of facemasks may influence physiological measurements, especially in prolonged clinical activities. Finally, future studies should include consideration of the type of dental procedure performed as well as a psychological assessment of the participants to control potential differences for these variables.

## 5. Conclusions

In the present study, the continuous use of an N95 in addition to a surgical facemask did not show a statistically significant effect on SpO_2_ during routine care; however, the concurrent use of an N95 and surgical facemask may be accompanied by a decrease in HR.

## Figures and Tables

**Table 1:** Difference in SpO_2_ between participants wearing an N95 in addition to a surgical facemask and those wearing only a surgical facemask at different timepoints.

Timepoints	N95 + Surgical Mask Group (%, SD), N = 57	Surgical Mask Group (%, SD), N = 31	Mean Difference	*p* Value ^a^	95% CI
T0	97.7 ± 0.9	98.0 ± 0.7	−0.2705	0.150	−0.641, 0.099
T1	97.7 ± 0.8	98.1 ± 0.7	−0.3189	0.072	−0.667, 0.029
T2	97.8 ± 0.9	97.9 ± 0.8	−0.1313	0.493	−0.510, 0.248
T3	98.0 ± 0.7	98.1 ± 0.6	−0.0382	0.795	−0.329, 0.253
*p* value ^b^	0.069	0.683			

^a^ paired mean difference with independent *t* test; ^b^ repeated measures ANOVA with sphericity assumed; 95% CI: confidence interval of the difference of means; SpO_2_: oxygen saturation; T0: baseline, before starting the dental procedure; T1: at 30 min; T2: at 1 h; T3: at the end of the clinical activity (≥4 h).

**Table 2:** Difference in HR between participants wearing an N95 in addition to a surgical facemask and those wearing only a surgical facemask at different timepoints.

Timepoints	N95 + Surgical Mask Group (bpm, SD), N = 57	Surgical Mask Group (bpm, SD), N = 31	Mean Difference	*p* Value ^a^	95% CI
T0	87.9 ± 15.1	78.9 ± 13.6	9.0340	0.007 *	2.549, 15.518
T1	85.0 ± 15.2	78.9 ± 11.6	6.1319	0.054	−0.108, 12.371
T2	85.5 ± 13.7	78.1 ± 10.2	7.3594	0.010 *	1.770, 12.938
T3	83.7 ± 11.9	77.5 ± 9.5	6.2295	0.014 *	1.283, 11.175
*p* value ^b^	0.012 *	0.722			

^a^ paired mean difference with independent *t* test; ^b^ repeated measures ANOVA with sphericity assumed; 95% CI: confidence interval of the difference of means; bpm: beat per minute; SpO_2_: oxygen saturation; T0: baseline, before starting the dental procedure; T1: at 30 min; T2: at 1 h; T3: at the end of the clinical activity (≥4 h). * denotes a statistically significant difference.

## Data Availability

The authors confirm that the data supporting the findings of this study are available within the article.
